# Trading heat and hops for water: Dehydration effects on locomotor performance, thermal limits, and thermoregulatory behavior of a terrestrial toad

**DOI:** 10.1002/ece3.3219

**Published:** 2017-09-26

**Authors:** Rodolfo C. O. Anderson, Denis V. Andrade

**Affiliations:** ^1^ Departamento de Zoologia Instituto de Biociências Universidade Estadual Paulista (UNESP) Rio Claro São Paulo Brasil

**Keywords:** Bufonidae, critical temperatures, optimal temperatures, preferred body temperatures, tolerance range

## Abstract

Due to their highly permeable skin and ectothermy, terrestrial amphibians are challenged by compromises between water balance and body temperature regulation. The way in which such compromises are accommodated, under a range of temperatures and dehydration levels, impacts importantly the behavior and ecology of amphibians. Thus, using the terrestrial toad *Rhinella schneideri* as a model organism, the goals of this study were twofold. First, we determined how the thermal sensitivity of a centrally relevant trait—locomotion—was affected by dehydration. Secondly, we examined the effects of the same levels of dehydration on thermal preference and thermal tolerance. As dehydration becomes more severe, the optimal temperature for locomotor performance was lowered and performance breadth narrower. Similarly, dehydration was accompanied by a decrease in the thermal tolerance range. Such a decrease was caused by both an increase in the critical minimal temperature and a decrease in the thermal maximal temperature, with the latter changing more markedly. In general, our results show that the negative effects of dehydration on behavioral performance and thermal tolerance are, at least partially, counteracted by concurrent adjustments in thermal preference. We discuss some of the potential implications of this observation for the conservation of anuran amphibians.

## 1 INTRODUCTION

In common with other ectotherms, amphibians often engage in activities in temperatures that may not allow optimal performance. This may reflect either limitations in thermal niche availability and/or because thermoregulatory behavior may conflict with the activity being performed or with other concurrent activities (Huey & Slatkin 1976; Huey & Kingsolver 1989). Likewise, due to their vulnerability to evaporative water loss and environmental contingencies in water availability, many amphibians are active under variable hydration states (see Tracy *et al*. 2014). Both of these factors, hydration state and temperature, are known to profoundly affect the physiological performance and tolerance of amphibians (Jørgensen 1997; Navas, Gomes & Carvalho 2008). Indeed, as wet‐skinned ectotherms, amphibians, especially those with terrestrial habits, are particularly sensitive to alterations in body temperature (Carey 1978) and hydration states (Wygoda 1984; Tracy *et al*. 2014). Moreover, the interaction between these factors influences the mechanisms involved with the regulation of each of them. For example, thermal sensitive of behavioral performance can be affected by dehydration state (Preest & Pough 2003; Titon and Gomes, 2010), while dehydration state can cause changes in preferred temperature and thermal tolerance (Claussen 1977; Mitchell & Bergmann 2016). Therefore, terrestrial amphibians are particularly predisposed to experience important compromises between water balance and thermoregulation (Tracy 1976; Preest & Pough 1989; Titon and Gomes, 2010).

The influence of temperature and hydration state on behavioral performance can be investigated by determining thermal performance curves (TPCs) at different levels of body hydration (Huey & Stevenson 1979; Beuchat, Pough & Stewart 1984; Huey & Kingsolver 1989; Preest & Pough 1989; Angilletta, Huey & Frazier 2010). From these curves, one can extract a number of informative parameters that includes the following: the optimal temperature (*T*_o_) in which maximal performance is obtained; optimal thermal breadth, which informs the interval in which performance is kept above a given level (e.g., 80%, B_80_); and critical thermal limits (CT), which sets the limits within which the animal is able to perform. Thus, changes in *T*_o_ may indicate whether the optimal temperature for performance on a given trait is affected by dehydration level. Changes in thermal performance breadth inform how dehydration may affect the capacity of the animals to buffer their performance against variations in temperature, while changes in CT may reveal conflicting demands associated with thermal tolerance and water balance (Claussen 1977; Feder & Hofmann 1999; Plummer *et al*. 2003).

Besides its influence on behavioral performance and its thermal sensitivity, dehydration is also known to affect thermoregulatory behavior in amphibians. For example, toads exhibited a decrease in their preferred body temperature (*T*_pref_) associated with dehydration (Williams & Wygoda 1993) or even with the exposition to dry air (Malvin & Wood 1991). This dehydration‐driven hypothermic response is thought to be of functional and ecological relevance because the potential for evaporative water loss is diminished at low body temperatures (Bundy & Tracy 1977; Tracy *et al*. 1993; Mitchell & Bergmann 2016). On the other hand, changes in *T*_pref_ may compromise behavioral performance if discordant with changes in *T*_o_. Therefore, it becomes highly relevant to examine the concurrent changes in *T*_o_ and *T*_pref_ in response to hydration level. This approach may allow for the evaluation on how thermoregulatory behavior may be adjusted to accommodate for the expected detrimental effects of dehydration on behavioral performance (see Angilletta *et al*. 2003; Navas *et al*. 2008; Artacho *et al*. 2015).

**Figure 1:**
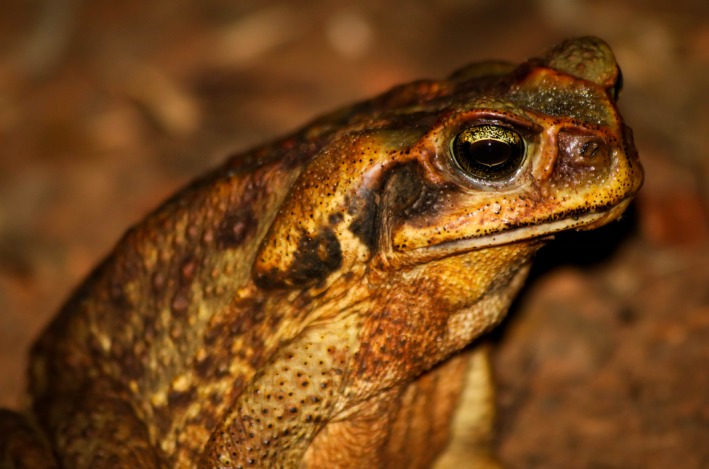
The toad *Rhinella schneideri*, a large‐bodied terrestrial anuran widely distributed in South America. Photograph by Lucas S. Almeida

The functional integration involving behavioral performance and its sensitivity to temperature, body temperature regulation, and hydration level constitutes a central aspect of ectotherms life history (Tracy 1976; Angilletta 2009). However, for many groups, such as terrestrial Neotropical anuran amphibians, such questions have rarely been examined (Navas *et al*. 2008). Accordingly, in the present study, we investigated the consequences of different hydration levels on the preferred body temperature and on the locomotor performance and its dependency to temperature in the terrestrial toad *Rhinella schneideri* (Figure 1). To this aim, we obtained thermal performance curves and determined the preferred body temperature on a thermal gradient for toads under different hydration levels. Based on the considerations made above, we predict that, as dehydration progresses, *T*_o_, *T*_pref_, thermal tolerance, and thermal performance breadth will decrease concurrently with a decrease in the absolute level of performance. *Rhinella schneideri* is a large‐bodied terrestrial toad widely distributed in South America from north and central Argentina, central Bolivia, Paraguay, Uruguay, and throughout Brazil. Along its distribution, *R. schneideri* occupies open and seasonally dry habitats, such as the Chaco and the Cerrado domains (Pramuk *et al*. 2008), being often found active away from water sources (Norman 1994; Santos *et al*. 2009). While many other sympatric anuran species estivate during cold and dry seasons, *R. schneideri* can remain active year around, even though with some seasonal decrease in activity (Noronha‐de‐Souza *et al*. 2015) and changes in thermoregulatory behavior (Bícego‐Nahas, Gargaglioni, & Branco 2001).

## 2 MATERIAL AND METHODS

### 2.1 Animals

We collected adult toads of both sexes in the municipality of Barbosa, state of São Paulo, Brazil (21.25048°S, 49.92132°W; datum: WGS84; elev. 371 m), on September 18 and 19, 2015. Although this period is within the reproductive season registered for *R. schneideri* (Norman 1994), no toad was calling or engaged in any other breeding behavior at the time of capture; instead, they were all found apparently foraging for food. After capture, animals were transported to the Laboratory of Comparative Animal Physiology, Universidade Estadual Paulista (UNESP), in the municipality of Rio Claro, state of São Paulo, Brazil, approximately 350 km from the collection site. In the laboratory, toads were maintained individually in plastic cages (20 × 30 × 70 cm) provided with shelter and a bowl full of water in a room with controlled temperature (23 ± 2°C) and natural photoperiod. We monitored toads daily and fed them crickets every other day, except 3 days prior to the experiments. The first trial began within 3–7 days after toads arrived at the laboratory, and 60 days was the total time until the end of all experiments. During this period in captivity, toad's body masses varied less than 3% from that measured at the time of capture.

### 2.2 Dehydration protocol

We randomly divided the toads into four experimental groups of ten individuals, each of these groups were submitted to a different level of hydration, varying from fully hydrated (100%) to animals dehydrated until they have lost 10% (90% hydrated), 20% (80% hydrated), or 30% (70% hydrated) of their initial fully hydrated body masses. The body masses at fully hydration were determined by placing individual toads in plastic containers (~20 cm of diameter) filled with 2 cm of distilled water, which allowed for the direct contact between the water and the toad's pelvic region. After 60 min under this condition, we emptied the urinary bladder by gently pressing the pelvic region and weighted the animals (± 0.01 g). The weights found under this protocol were accepted as the body mass at fully hydration. Following the determination of the fully hydrated body masses, except for 100%, we proceed to the dehydration protocol until the desired level of dehydration was attained. To promote dehydration, toads were placed individually in a wind tunnel (airflow of 2.5 ± 0.5 m/s), at 25°C and relative humidity of 50 ± 5%. Under these conditions, toads lost water by average of 10 g/hr and were weighted every 15 min until they had attained the desired hydration level. All experiments started immediately thereafter.

Except for the control group, each individual toad was submitted to the dehydration procedure for eight times along the duration of the study. Each individual was always dehydrated to the same level, which was dictated by its allocation within a given experimental group. After the first dehydration, animals were submitted to the protocol for *T*_pref_ determination; from the second to the sixth dehydration bouts, toads were submitted to the locomotor performance trials, five in total, and after the seventh and the eight dehydration bouts, they were subjected to the measurement of the minimal and maximal critical temperatures, respectively (see details for each protocol below). Between each dehydration procedure, animals returned to their maintenance cages where they had free access to water, were fed, and were allowed to recover for a minimum period of 3 days.

### 2.3 Preferred temperature

Preferred body temperature was determined in a circular arena (90 cm diameter and 100 cm height) with walls built with galvanized steel plates and floor mounted on a copper plate. In this arena, we produced a thermal gradient that ranged from 13 to 40°C. This was achieved by the use of heat tapes (reptile heat tape 6″, THG Heat Tapes) secured on the external side of the copper plate on one side of the arena and by the placement of packs of artificial ice (GeloTech, model 700), also on the external side of the copper plate, on the opposite side of the arena. The arena floor was covered with a black nonwoven fabric (TNT, Temasi), which was discarded and replaced after each individual measurement. The gradient temperature distribution and its evenness were checked every 30 min using an infrared thermal camera (Flir SC‐640; Flir Systems Inc.).

Before the beginning of *T*_pref_ measurements, toads were kept inside a climatic chamber (122FC Eletrolab) at 20°C for 2 hr to ensure that all individuals had the same body temperatures at the onset of the experiments. Next, we weighted the animals (Marte AS5500C, ± 0.01 g) and measured their cloacal (Hand Held Digital Thermometer, ETI Thermometers) and dorsal (infrared thermometer ETI—EcoTemp model) temperatures. After that, toads were individually released into the middle area of the thermal gradient where they were left undisturbed for 30 min. Following this accustomization period, we measured the superficial dorsal temperature (infrared thermometer ETI—EcoTemp model) of the toads every 15 min for ten times. Finished this period, we immediately measured the cloacal temperature and again weighed the animals (same instruments as above). All trials were conducted between 18:00 and 00:00, when toads were active, in a room with dim light and air temperature controlled at 25 ± 2°C. No toad lost more than 1% of their initial body mass during any of the trials.

### 2.4 Locomotor performance

For the determination of the thermal performance curves, we focused on locomotor performance because foraging, reproduction, escape from predators, and many other ecologically relevant activities are inextricably associated with locomotion in anuran amphibians (Prates *et al*. 2013 and references therein). Moreover, previous studies found that locomotion may be greatly influenced by temperature (Rome, Stevens, & John‐Alder 1992) and dehydration (Preest & Pough 1989) in amphibians. Therefore, herein, we proceed to test the locomotor performance of our toads in five different temperatures (15, 20, 25, 30, and 35°C), in random order and performed on different days, usually more than 3 days apart to each other.

Locomotor performance was measured in a circular track made of polyethylene (15 cm width, 80 cm height, and 150 cm diameter) located inside a climatized room (Fitotron 011—Eletrolab) set to the desired temperature level. Previous to each trial, toads were kept for a period of 2 hr in a cage inside a smaller climatic chamber (122FC Eletrolab) set to the same temperature they were going to be tested. After this period, toads were weighed and had their cloacal temperature measured. Next, they were placed individually into the test track and the performance trial immediately begun. During the trial, toads were continuously stimulated to move by touches of a wood rod on the urostile during a 10 min' period. The distance covered during this period was defined as the absolute locomotor performance of the animal being tested. By the end of the trial, the cloacal temperature and body mass were once again recorded. No toad showed a change in body temperature more than 1.5°C apart from the designated experimental temperature and did not lose more than 1% of their initial body mass during any of the trials.

### 2.5 Critical temperatures

To determine maximum and minimal critical temperatures, toads were placed individually in plastic containers kept inside a climatic chamber (122FC Eletrolab) at 23°C. From this initial temperature, we either cooled or heated the chamber at a rate of 1°C/10 min. CT endpoints were established as the temperature in which toads lost their ability to right themselves, within 15 s, when manually turned upside down. During the experiment, this righting response was initially verified every 10 min, but below 15°C and above 35°C, it was checked every minute. Immediately after the loss of the righting reflex, we measured the body temperature of the toads by recording their cloacal temperature (Hand Held Digital Thermometer, ETI Thermometers).

After the experiments, we immersed the toads inside bowls filled with water at ambient temperature for recovering. If the toad died following the experiments, we discarded its data from the analyses. This happened only in three instances for the measurements of CT_max_, twice at the 70% hydration level, and once at 80%.

### 2.6 Data analyses

For each individual toad, we defined *T*_pref_ as the median skin body temperatures recorded at the thermal gradient. We also used the 25th and 75th quartiles of these same temperature recordings to establish the lower and upper limits of the preferred temperature range, respectively (Hertz, Huey & Stevenson 1993).

Locomotor performance was initially relativized as a percentage of the maximum absolute performance attained by each individual toad at any of the temperatures tested. After, the relativized levels of locomotor performance were combined with lower and upper performance bounds (*i.e*., performance = 0) taken from the critical temperature determinations, and plotted against temperature. Over these data, thermal performance curves were adjusted using the software TableCurve 2D (Systat Software) (see Angilletta 2006). In all cases, we applied LogNormal function, which provided the best adjustment (*R* ^2^ > .95) for describing the TPCs. We established the thermal optimum (*T*_o_) as the maximum (peak) of the curve (i.e., relative performance = 100%) and the thermal performance breadth as the temperature interval within which the toads reached 80% (lower B80 and upper B80) of their maximum performance (see Huey & Stevenson 1979; Tracy *et al*. 1993; Angilletta, Niewiarowski & Navas 2002). Also, we calculated the B80 range as the difference between upper B80 and lower B80.

Differences among hydration levels for all the parameters examined were tested with an analysis of variance (ANOVA). Previously, we checked for the potential influence of body masses variation on each and every multi‐group comparison using an analyses of covariance (ANCOVA). However, since no effect of body mass was found in any case, we proceeded with the ANOVA test. Whenever, statistical differences were revealed by the ANOVA test, they were isolated by the Student ‐ Newman ‐ Keuls (SNK) test. In cases in which our data failed to attend the premises of normality and homoscedasticity of variance, we employed the Kruskal‐Wallis test, followed by Dunn´s test, whenever necessary.

We used a Linear Mixed‐effects Model (*lme4* package) to examine the effects of temperature and dehydration on the absolute locomotor performance of *R. schneideri*. We set the dehydration levels and temperature as factors and the absolute locomotor performance as the response variable. As the same individuals were tested repeteadly in different temperatures, we set the individuals as random factors. Finally, we performed multiple comparisons of means (Tukey contrasts) post hoc test for both temperature and dehydration level (package *multcomp*).

All the analyses were realized using R version 3.2.3 (R Development Core Team, 2015), and differences were accepted as significant at the level of *p *< 0.05.

## 3 RESULTS

### 3.1 Body mass

Toads keep their body condition along the data collection period and their body mass before dehydration did not differ among experimental groups (Kruskal–Wallis one‐way *H* = 2.530; *df* = 3; *p *=* *.47; Table 1) and neither among different trials (Kruskal–Wallis one‐way *H* = 1.521; *df* = 3; *p *=* *.678). Changes in body mass due to the dehydration protocol only reflected the fact that the different experimental groups were subjected to different levels of loss in body water content and were not considered as a factor in any of our latter analyses.

**Table 1:** Standard body mass of *Rhinella schneideri* for the different experimental groups. The body mass values presented correspond to the mass of the toads before being dehydrated to their designated dehydration level

Dehydration level (%)	*N*	Mean (g)	*SD* (g)	Range (g)
100	10	178.39	99.34	84.11–364.4
90	10	131.61	64.36	82.42–239.72
80	10	148.46	92.73	80.69–339.95
70	10	142.43	63.28	78.82–268.21

### 3.2 Preferred body temperature

**Figure 2:**
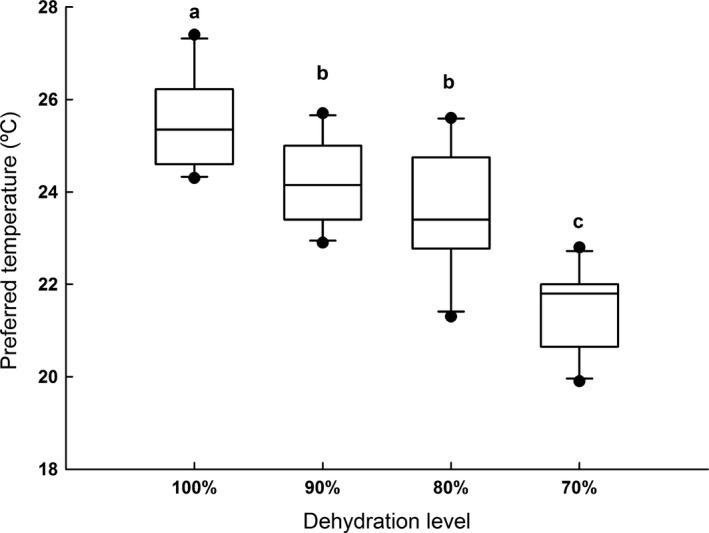
Influence of dehydration level on the preferred body temperatures (*T*_pref_), of *Rhinella schneideri*. The line inside the box plot, the borders, and the whiskers represent, respectively, the median, the second and third interquartile range, and the range of the data; dots are outliers. Different letters above boxplots indicate significant differences

*T*_pref_ differed among the different hydration levels (*F*_1,36_ = 69.803; *p *<* *.001), with fully hydrated toads exhibiting *T*_pref_ values higher than all other hydration levels. On the opposite, the most dehydrated toads of the 70% group had the lowest *T*_pref_ among all experimental groups of fully hydrated toads (*p *<* *.001 in all comparisons; Figure 2), while no significant difference was detected between 90% and 80% (*p *=* *.199; Table 2; Figure 2).

**Table 2:** Preferred temperature (*T*_pref_), thermal limits (CT_max_ and CT_min_), optimal temperature (*T*_o_), thermal performance breadth (lower and upper B80), B80 range, and thermal tolerance range (TR) of *Rhinella schneideri* at different levels of dehydration. The values are show as mean ± standard deviation

	Dehydration level			
	100%	90%	80%	70%
*T*_pref_ (°C)	25.49 ± 0.98	24.24 ± 0.9	23.63 ± 1.33	21.46 ± 0.88
CT_min_ (°C)	6.37 ± 1.04	6.36 ± 0.87	9.38 ± 1.14	9.77 ± 1.57
CT_max_ (°C)	40.36 ± 0.84	39.82 ± 0.34	38.34 ± 0.7	38.5 ± 0.46
*T*_O_ (°C)	30.1 ± 1.76	28.29 ± 1.85	23.34 ± 1.04	23.3 ± 0.81
B80 (°C)	22.1–35.78	20.45–34.47	17.23–30.32	17.44–30.4
B80 range (°C)	16.76 ± 2.67	14.35 ± 3.58	10.38 ± 3.98	11.42 ± 2.11
TR (°C)	33.98 ± 1.62	33.33 ± 0.84	29.03 ± 0.88	28.72 ± 1.72

### 3.3 Absolute locomotor performance

**Figure 3:**
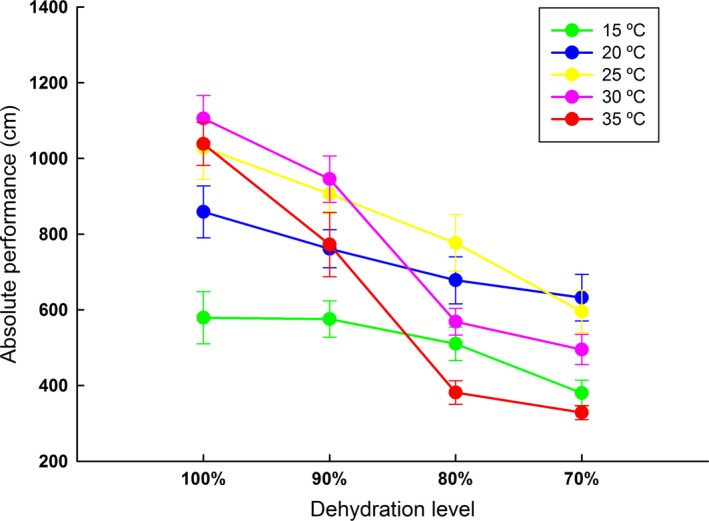
Absolute locomotor performance of *Rhinella schneideri*, at different levels of dehydration, at five different temperatures. Each symbol represents the mean and the associated whiskers the confidence interval (95%)

Temperature (F = 304.60; *df* = 4; *p *<* *.001), dehydration (F = 99.45; *df* = 3; *p *<* *.001), and the interaction of these variables (F = 22.34; *df* = 12; *p *<* *.001) strongly affect the absolute locomotor performance of *R. schneideri* (Table S1). In general, the locomotor performance improved with temperature elevation (Figure 3; Table S2). The maximum distances covered by toads lowered with the decrease in hydration level (Figure 3; Tables S2 and S4) and this effect was more pronounced at warmer temperatures (Figure 3; Table S1 and S3).

### 3.4 Optimal temperatures and thermal performance breadth

**Figure 4:**
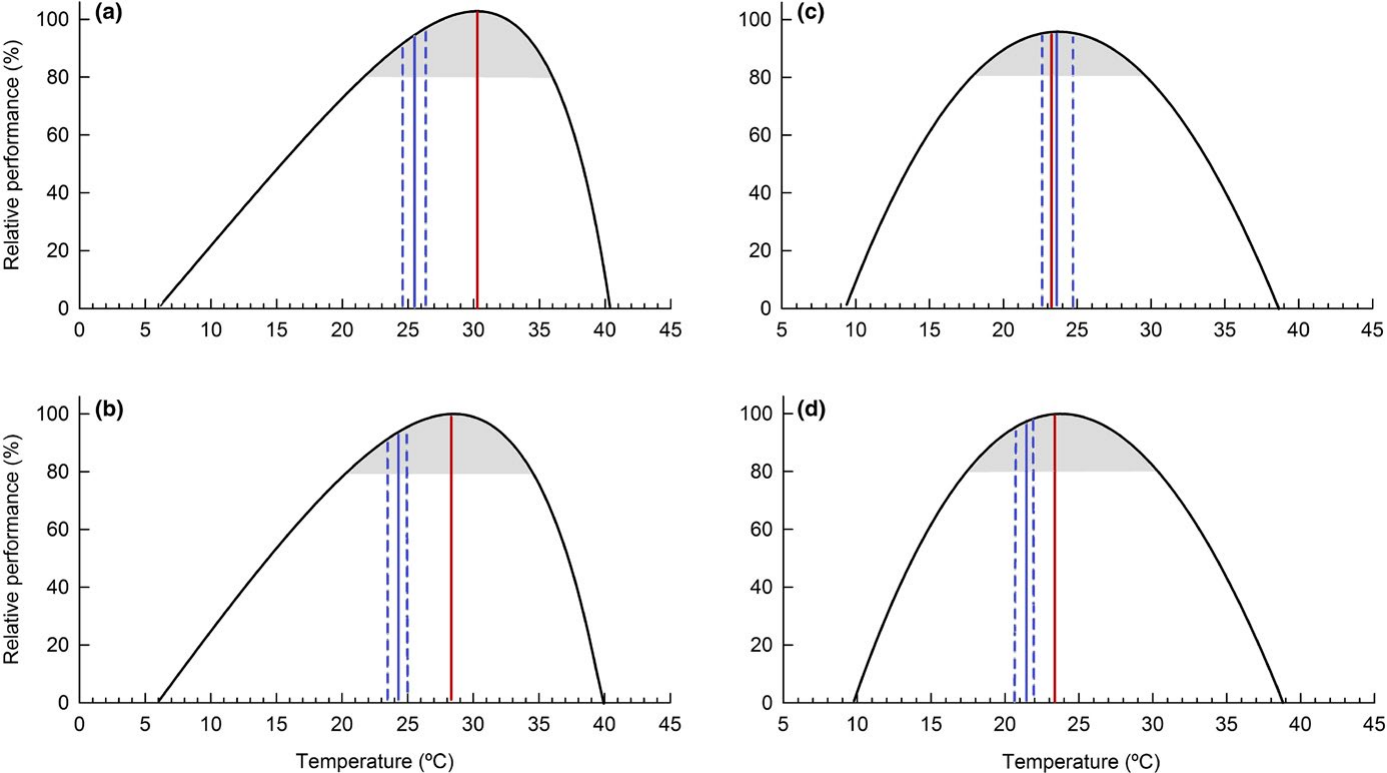
Thermal performance curves of *Rhinella schneideri* at four different levels of dehydration (a = 100%; b = 90%; c = 80%; d = 70%). The red vertical line represents the optimal temperature, the gray area represents the thermal performance breadth (80% of maximal performance), the blue solid vertical line represents the mean *T*_pref_, and the blue dotted lines represent the *T*_pref_ interquartile range (25th and 75th)

**Figure 5:**
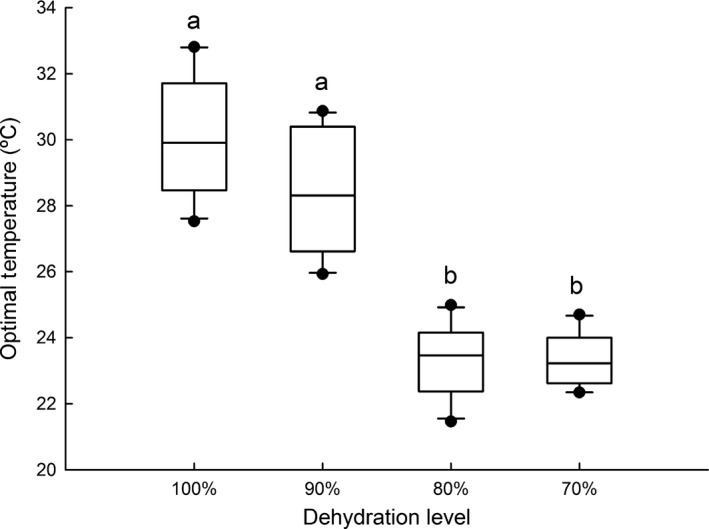
Optimal temperature for locomotor performance (*T*_o_) of *Rhinella schneideri* at different levels of dehydration. The line inside the box plot, the borders, and the whiskers represent, respectively, the median, the second and third interquartile range, and the range of the data; points are outliers. Different letters above boxplots indicate significant differences

**Figure 6:**
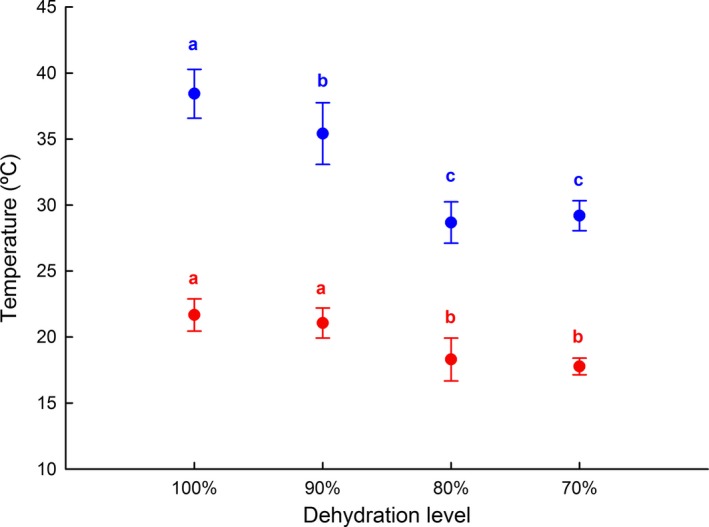
Upper B80 (blue) and lower B80 (red) values for *Rhinella schneideri* at four different dehydration levels. The dots represent the mean and the whiskers the confidence interval (95%). Different letters above boxplots indicate significant differences

The optimal temperature for locomotor performance was significantly affected by dehydration (*H* = 29.983; d.f = 3; *p *<* *.001) with toads 100% and 90% hydrated exhibiting higher *T*_o_s in comparison with those at 80% and 70% (Table 2; Figures 4 and 5). Lower B80 was greater (*i.e*., warmer) in better hydrated toads (100% and 90%) than in those more dehydrated (80% and 70%) (Table 2; Figure 6; *H* = 22.804; *df* = 3; *p *<* *.001). Upper B80 were greater (*i.e*., warmer) at higher hydration levels, except between 80% and 70% (*F*_3,39_ = 63.29; *p *<* *.001; Figure 6). As a consequence, B80 range was broader for toads 100% and 90% hydrated in comparison with those at the 80% and 70% hydration levels (Table 2; Figure 4; *H* = 17.77; *df* = 3; *p *<* *.001).

### 3.5 Critical temperatures

**Figure 7:**
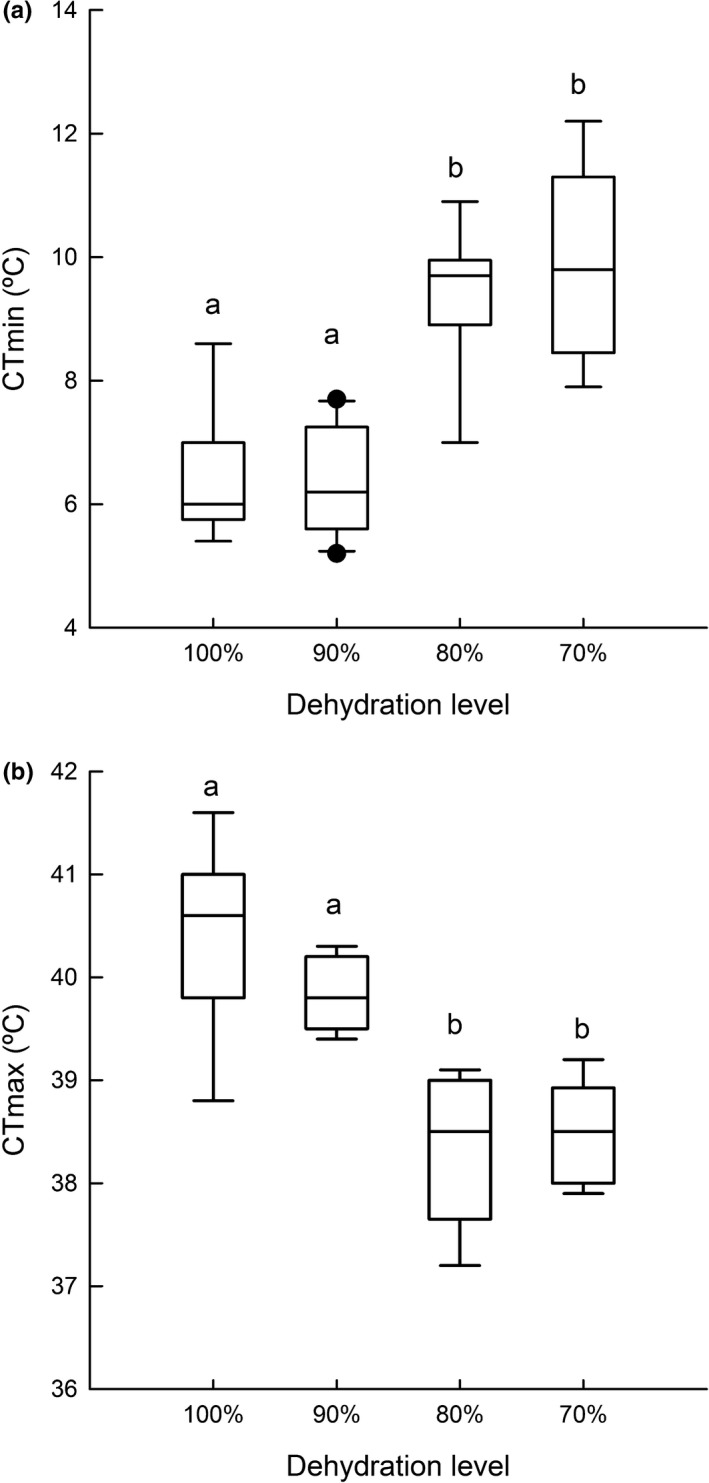
Critical thermal minimum (a) and maximum (b) of *Rhinella schneideri* at different levels of dehydration. The line inside the box plot, the borders, and the whiskers represent, respectively, the median, the second and third interquartile range, and the range of the data; dots are outliers. Different letters above boxplots indicate significant differences

**Figure 8:**
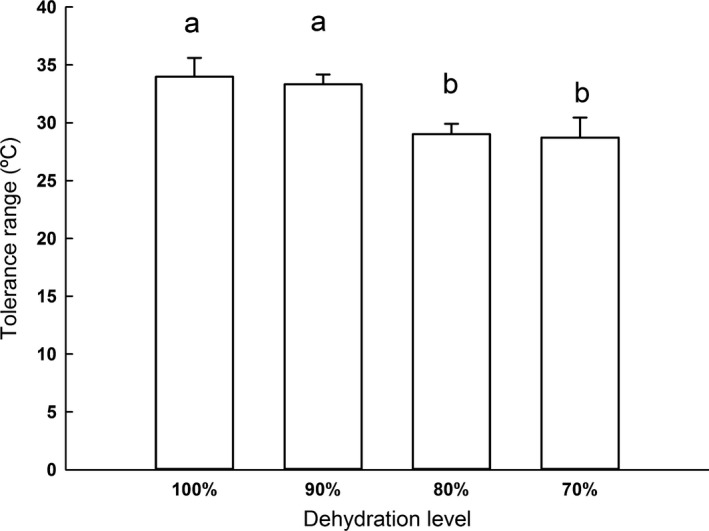
Mean tolerance range of *Rhinella schneideri* at different levels of dehydration. The whiskers and letters above the bars represent the standard deviation and significant differences, respectively

The CT_min_ (*F*_3,36_ = 38.17; *p *<* *.001) and CT_max_ (*F*_3,36_ = 36.14; *p *<* *.001) were both affected by dehydration (Figure 7; Table 2). More specifically, CT_min_ and CT_max_ for the groups 90% and fully hydrated were higher and lower, respectively, compared to the less hydrated groups of 80% and 70%. Related to the changes in CT limits, tolerance range was found to be broader for 90% and fully hydrated groups in comparison with the 80% and 70% ones (Figure 8; *F*_3,33_ = 36.979; *p *<* *.001).

## 4 DISCUSSION

Temperature and dehydration affected the locomotor performance of *R. schneideri*, as previously reported for other terrestrial anurans (Moore & Gatten 1989; Preest & Pough 1989), including congeneric species (Titon et al., 2010; Prates *et al*. 2013; Titon and Gomes, 2010). Our results clearly showed that the better hydrated the animal, the better was their absolute locomotor performance in all temperatures tested. This deleterious influence of dehydration on the toad's performance, however, was temperature dependent. In general, the drop in performance associated with dehydration was more pronounced at higher temperatures (see Figure 1). Thus, if on the one hand higher temperatures promote a better performance, which possibly will translate in positive effects on fitness (Huey & Kingsolver 1989; Angilletta *et al*. 2010), on the other hand, higher temperatures associated with low relativity humidity increase the potential for water evaporation, which may culminate in dehydration that, as we found, had the most severe consequences at higher temperatures. Such potential trade‐off may be of limited importance if water availability is plenty and animals can be active at optimal temperatures without compromising their osmotic balance. In cases in which water availability is limited and dehydration is unavoidable, toads may remedy the situation by changing thermal preference to cooler levels and/or become less active (see Discussion below). However, if elevated temperature occurs in combination with limited water availability, our results indicate that this condition may translate into severe consequences to organismal performance. Within the *Rhinella* genus, such consequences seem to vary with interspecific differences in the thermal sensitivity of performance to dehydration, which, in turn, may be related to differences in the macroclimatic attributes of the different habitats in which these toads occur (see Titon and Gomes, 2010). Whatever the case, the increased frequency of extreme climatic events (Dillon *et al*. 2016; Buckley & Huey 2016a; Sheldon & Dillon 2016) combining higher temperatures with dry conditions (Camacho, Rodrigues & Navas 2015; Buckley & Huey 2016b; Williams *et al*. 2016) may pose an important threat to amphibian conservation.

Optimal temperatures for locomotor performance (*T*_o_) and performance breadth (B80) both decreased with dehydration. These results suggest that the underlying processes supporting locomotion changed their thermal sensitivity with dehydration (see also McClanahan 1964; Hillman 1980; Hillman 1987). In general, the deleterious effects of dehydration were accentuated at higher temperatures and, as result, the optimal level of performance moved toward low temperatures. Considering that the combination of high temperatures and dehydration does not only compromise performance importantly but also pose major risks for water balance (discussed above), the attainment of optimal levels of performance at lower temperatures when dehydration becomes severe may be interpreted as important to decrease the risks of excessive evaporative water loss without compromising performance too heavily. For this to happen, concurrent changes in thermal preference (discussed below) are instrumental. In fact, dehydration may add to the importance of thermoregulatory adjustments, as the narrowing in B80 indicates that the buffering capacity to sustain performance against temperature variation was also compromised with dehydration. Finally, changes in thermal behavior are contingent to the availability of adequate thermal niches and, therefore, human‐induced changes in habitat attributes may hinder such organismal response.

We found that thermal tolerance declined with hydration level in *R. schneideri*. This effect was particularly prominent for the more dehydrated groups and included both an increment in CT_min_ and a decrease in CT_max_. These results suggest the existence of a conflicting demand between water homeostasis and thermal tolerance. Under a water deficit situation, amphibians experience considerable rises in their body fluid concentrations and can also mobilize different metabolites thought to counteract dehydration damages (Shoemaker 1964; Degani & Warburg 1984; Jørgensen 1997; Anderson *et al*. 2017). Similarly, the exposure to extreme temperatures, both cold and warm, induces the cellular production of molecules in order to mitigate thermal damages (*e.g*., heat‐shock proteins) (Easton, Rutledge, & Spotila 1987; Feder and Hofmann, 1999, see also Ketola‐Pirie & Atkinson 1983). Therefore, dehydration and thermal stress trigger molecular pathways and/or cause biochemical alterations that may interfere with each other response and, possibly, limit the magnitude of tolerance. Finally, as dehydration is accompanied by a decrease in the rates of evaporative water loss in *R. schneideri* (Anderson *et al*. 2017), the buffering of evaporative cooling against body temperature elevation might be compromised on dehydrated toads, which will lead to a decrease in CT_max_. All these ideas agree with our observation that as dehydration becomes more severe, thermal tolerance range is narrowed.

Fully hydrated toads attained maximum levels of performance at temperatures much higher than their preferred body temperatures (Figure 4). Similar mismatches have been previously reported (Tracy *et al*. 1993; Köhler *et al*. 2011; Mitchell and Bergmann, 2016; Gvoždík & Kristín 2017) and may involve functional constraints and, perhaps, methodological limitations. For example, we should consider that the underlying processes driving thermoregulatory behavior during the determination of *T*_pref_ in a thermal gradient are, most likely, diverse from those at play during the assessment of the sensibility of locomotor performance to temperature (see also Gvoždík & Kristín 2017). Also, *T*_o_ determination usually is based on the assessment of a single trait, for example, locomotion (*e.g*., present study), while thermal preference may reflect the integration of various simultaneous processes (Huey & Stevenson 1979; Angilletta, Niewiarowski & Navas 2002; Martin & Huey 2008; Navas *et al*. 2008). The mismatch between *T*_pref_ and *T*_o_ may also result from differences in the selective forces that have acted during the evolution of each of these traits, as well as differences in how conservative they are. In such case, historical factors may contribute to the incongruence observed between *T*_pref_ and *T*_o_ (Huey & Bennett 1987; Angilletta, Hill & Robson 2002; Martin & Huey 2008; Mitchell & Bergmann 2016; Gvoždík & Kristín 2017).

Dehydration shifted thermal preference toward lower body temperatures in *Rhinella schneideri*. Therefore, the change in thermal preference agreed with the ideas discussed above in terms of accommodating conflicting demands related to water balance and behavioral performance. In terms of water balance, the decrease in thermal preference with dehydration is thought to be of great functional and ecological relevance as lower temperatures diminish the potential for water evaporation. Combined with the decrease in evaporative water loss associated with dehydration (Anderson *et al*. 2017), these responses are likely to assist animals already under a hydric deficit to slow down the rate of evaporative water loss, and eventually, reestablish their normal water balance (see also Bundy & Tracy 1977; Malvin & Wood 1991; Tracy *et al*. 1993; Williams & Wygoda 1993; Mitchell and Bergmann, 2016). In terms of behavioral performance, the concurrent changes in *T*_pref_ and *T*_o_ reduced the mismatch between these two variables (discussed above) as dehydration became more severe. Therefore, even if operating at low absolute levels under dehydration, the performance in locomotion could still occur at its optimum, as long as the animals were allowed to adjust their thermal preference. Such response has been previously reported for other anuran species (Köhler *et al*. 2011; Mitchell and Bergmann, 2016) and may contribute importantly to mitigate the effects of dehydration on homeostasis (Preest & Pough 1989; Preest & Pough 2003; Gvoždík & Kristín 2017).

In summary, our results show that while dehydration was associated with negative effects on behavioral performance and thermal tolerance, concurrent changes in thermoregulatory behavior contributed to minimize these effects. The most severe consequences of dehydration, not surprisingly, were associated with high temperatures. These observations highlight the importance of the availability of adequate thermal niches in order to allow the amphibians to resort on thermoregulatory adjustments in response to water stress situations. In this regard, future scenarios in which the combination of higher temperatures and low water availability is predicted to become more frequent (McMenamin, Hadly & Wright 2008; Hof *et al*. 2011; Sherwood & Fu 2014; Sunday *et al*. 2014) may pose quite significant threats to the conservation of terrestrial amphibians (Todgham & Stillman 2013; Sunday *et al*. 2014; Nowakowski *et al*. 2016).

## CONFLICT OF INTEREST

The authors declare no conflict of interest.

## AUTHORS' CONTRIBUTIONS

RCOA and DVA conceived the ideas and designed methodology; RCOA collected and analyzed the data; RCOA and DVA contributed equally to the writing of the manuscript. All authors contributed critically to the drafts and gave final approval for publication.

## DATA ACCESSIBILITY

Data will be deposited in the Dryad Digital Repository.
