# Supplementary material for: Trading heat and hops for water: Dehydration effects on locomotor performance, thermal limits, and thermoregulatory behavior of a terrestrial toad
Source: Ecol Evol. 2017 Sep 26;7(21):9066–75. doi: 10.1002/ece3.3219 (PMC5677477; doi:10.1002/ece3.3219)
Supplement: Supplementary file 1 [file ECE3-7-9066-s001.docx]

Table A1. Output of GLM analyses showing the effect of dehydration, temperatures and its interaction on the locomotor performance of *Rhinella schneideri.*

|  | Estimate | Standard Error | t value | *P* |
| --- | --- | --- | --- | --- |
| Dehydration | -10.631 | 3.26 | -3.252 | < 0.001 |
| Temperature | -82.052 | 10.78 | -7.607 | <0.01 |
| Interaction | 1.03 | 0.12 | 8.204 | <0.001 |

Null deviance : 11289188 on 199 degrees of freedom; residual deviance: 3877943 on 196 degrees of freedom.

Table A2. Summary of GLM analyses showing the effect of temperature, dehydration level and the interaction of these factors in the absolute locomotor performance of *Rhinella schneideri*. In bold are represented the terms expected to influence the model. desid: dehydration level; temp: temperature tested.

|  | *Estimated* | *Std. Error* | *t value* | *P* |
| --- | --- | --- | --- | --- |
| intercept | **380.00** | **25.33** | **15.003** | **<0.0001** |
| desid80 | **130.00** | **35.82** | **3.629** | **<0.0001** |
| desid90 | **195.50** | **35.82** | **5.458** | **<0.0001** |
| desid100 | **199.00** | **35.82** | **5.556** | **<0.0001** |
| temp20 | **252.00** | **35.82** | **7.035** | **<0.0001** |
| temp25 | **215.50** | **35.82** | **6.016** | **<0.0001** |
| temp30 | **115.00** | **35.82** | **3.211** | **<0.001** |
| temp35 | -51.50 | 35.82 | -1.438 | 0.15224 |
| desid80:temp20 | -84.00 | 50.66 | -1.658 | 0.09902 |
| desid90:temp20 | -66.00 | 50.66 | -1.303 | 0.19428 |
| desid100:temp20 | 28.00 | 50.66 | 0.553 | 0.58113 |
| desid80:temp25 | 51.00 | 50.66 | 1.007 | 0.31540 |
| desid90:temp25 | **115.50** | **50.66** | **2.280** | **0.02378** |
| desid100:temp25 | **234.50** | **50.66** | **4.629** | **<0.0001** |
| desid80:temp30 | -56.50 | 50.66 | -1.115 | 0.26619 |
| desid90:temp30 | **255.00** | **50.66** | **5.034** | **<0.0001** |
| desid100:temp30 | **411.50** | **50.66** | **8.123** | **<0.0001** |
| desid80:temp35 | -77.00 | 50.66 | -1.520 | 0.13026 |
| desid90:temp35 | **248.50** | **50.66** | **4.906** | **<0.0001** |
| desid100:temp35 | **511.00** | **50.66** | **10.087** | **<0.0001** |

Null deviance : 11289188 on 199 degrees of freedom; residual deviance: 3877943 on 196 degrees of freedom.

Table A3. Multiple comparisons of means (Tukey Contrasts) for a simultaneous test for general linear hypotheses of the temperatures influencing locomotor performance of *Rhinella schneideri*. In bold are represented the terms expected to influence the model

| *Temperatures* | *Estimated* | *Std. Error* | *z value* | *P* |
| --- | --- | --- | --- | --- |
| 20 – 15 = 0 | **252** | **35.82** | **7.035** | **<0.001** |
| 25 – 15 = 0 | **215.75** | **35.82** | **6.016** | **<0.001** |
| 30 – 15 = 0 | **115** | **35.82** | **3.211** | **0.011** |
| 35 – 15 = 0 | -51.5 | 35.82 | -1.438 | 0.603 |
| 25 – 20 = 0 | -36.5 | 35.82 | -1.019 | 0.84 |
| 30 – 20 =0 | **-137** | **35.82** | **-3.825** | **0.0013** |
| 35 – 20 = 0 | -303.5 | **35.82** | **-8.473** | **<0.001** |
| 30 – 25 = 0 | **-100.5** | **35.82** | **-2.806** | **0.0402** |
| 35 – 25 = 0 | **-267** | **35.82** | **-7.454** | **<0.001** |
| 35 – 30 = 0 | **-166.5** | **35.82** | **-4.648** | **<0.001** |

Table A4. Multiple comparisons of means (Tukey Contrasts) for a simultaneous test for general linear hypotheses of dehydration levels influencing locomotor performance of *Rhinella schneideri*. In bold are represented the terms expected to influence the model

| *Dehydration level* | *Estimated* | *Std. Error* | *z value* | *P* |
| --- | --- | --- | --- | --- |
| 80 – 70 = 0 | **130** | **35.82** | **3.629** | **<0.001** |
| 90 – 70 = 0 | **105.5** | **35.82** | **5.458** | **<0.001** |
| 100 – 70 = 0 | **199** | **35.82** | **5.556** | **<0.001** |
| 90 – 80 = 0 | 65.5 | 35.82 | 1.829 | 0.259 |
| 100 – 80 = 0 | 69 | 35.82 | 1.926 | 0.216 |
| 100 – 90 =0 | 3.5 | 35.82 | 0.098 | 0.999 |
